# Coping With the Fear of Compartment Syndrome Without Compromising Analgesia: A Narrative Review

**DOI:** 10.7759/cureus.30776

**Published:** 2022-10-27

**Authors:** Kartik Sonawane, Preethi Dhamotharan, Hrudini Dixit, Palanichamy Gurumoorthi

**Affiliations:** 1 Anesthesiology, Ganga Medical Centre and Hospitals Pvt. Ltd., Coimbatore, IND; 2 Anesthesiology and Perioperative Medicine, Ganga Medical Centre and Hospitals Pvt. Ltd., Coimbatore, IND; 3 Anesthesiology, Sir H. N. Reliance Foundation Hospital and Research Centre, Mumbai, IND

**Keywords:** ultrasound-guided regional anesthesia, acute pain, postoperative pain relief, below-knee surgeries, lower limb trauma, orthopedic anesthesia, regional anesthesia and chronic pain, acute exertional osteofascial compartment syndrome, compartment syndrome leg, perioperative pain management

## Abstract

Pain management in trauma or surgery with a high risk of developing compartment syndrome (CS) is always challenging due to fears of masking symptoms that could delay diagnosis and treatment. Regional anesthesia/analgesia (RA) can facilitate enhanced postoperative recovery and improve patient satisfaction by providing excellent postoperative analgesia. However, its consideration in surgeries with a high risk of developing CS remains controversial and contentious. Studies suggest focusing more on early diagnosis through regular vigilant monitoring with a high index of suspicion rather than discontinuing the analgesic method alone. The most consistent features in all reported cases of CS were altered sensation in the affected limb, disproportionate pain in the presence of a functional nerve block, and an escalating need for analgesics.

Several extrinsic or intrinsic factors are responsible for the progressive increase in compartment pressure that can lead to vascular compromise and subsequent ischemic changes in muscles, tissues, and nerves. Measurement of intracompartmental pressure (ICP) has always been considered the gold standard for diagnosing CS. An ICP of 30 mm Hg is considered the cut-off point for fasciotomy that helps restore muscle perfusion and avoid irreversible tissue damage. The chronology of symptoms can sometimes provide clues to the severity of CS, the pathophysiology involved, and the management required. Therefore, it is necessary to look for warning signs, further investigate the causes, and make quick decisions to diagnose and treat CS and its complications on time. Any delay in the diagnosis and treatment of CS can result in high morbidity and poor outcomes. A well-integrated interprofessional team of health professionals can deliver the required complexity of care through a holistic and multidisciplinary approach.

This review article highlights the symptoms, risk factors, and pathophysiology involved in CS. It can guide readers in choosing various options to diagnose, prevent, and treat CS. It also discusses the role of RA in patients or surgeries prone to developing CS.

## Introduction and background

All skeletal muscles in the human body are enclosed in a fascial sheath, a thin, inelastic layer of connective tissue that limits the ability to expand rapidly [1]. A rise in pressure of this closed osteofascial compartment leads to vascular impairment that causes decreased oxygen and essential nutrients delivery, resulting in local hypoxia and irreversible tissue damage. Such a clinical condition is known as compartment syndrome (CS), which can be acute or chronic depending on the duration. Acute CS (ACS) is considered an emergency condition [2] that can potentially lead to ischemia and necrosis, resulting in significant morbidity and mortality. Chronic CS is also known as exertional CS due to its association with excessive exertion during sports or strenuous physical activity [3-5]. Pseudo-CS results from partial vascular occlusion in the lower limb [6]. CS without limb involvement is known as well-leg CS, seen in abdominal surgery [7].

CS can develop in various anatomical compartments from head to toe, including the arm, forearm, hand, abdomen, gluteal region, thigh, leg (tibia), and foot [8-14]. However, it is more common in the lower limbs. The incidence of ACS is 1-9% of all leg fractures, of which 75% of ACS cases [15] are often associated with long bone fracture (predominantly tibial diaphysis) and 18% of the cases with distal radius fracture [14,16-18]. Among the four compartments of the leg (anterior, lateral, deep posterior, and superficial posterior), the anterior (extensor) compartment is the most common location for CS [19]. Increased compartmental pressure can be associated with the compression of nerves present in each compartment, namely, the deep peroneal nerve (anterior), superficial peroneal nerve (lateral), saphenous nerve (superficial posterior), and tibial nerve (deep posterior). The risk of developing CS in open tibial fractures is more frequent (6%) than in closed tibial fractures (1-2%) [13]. The risk is even higher in cases of comminuted fractures [14,17]. Non-fracture soft-tissue injuries account for about 20% of ACS cases [20].

This review article highlights the symptoms, risk factors, and pathophysiological processes associated with CS and describes various options to diagnose, avoid, and treat CS. It also discusses the role of regional anesthesia (RA) in patients or surgeries prone to developing CS.

## Review

This narrative review describes the pathophysiology, diagnosis, and treatment of CS; available pain management strategies; and the role of RA in such cases. Related literature searches were performed using online platforms (PubMed, Medline and Embase databases, Cochrane Library, and Google Scholar) using relevant search terms (compartment syndrome/CS/diagnosis/pathophysiology/risk factors/pain management/ epidural/analgesia/an(a)esthesia/an(a)esthetic/nerve block/regional/diagnosis/surgery). Articles published in English were selected, and their reference sections were hand-searched for additional information.

Responsible factors for compartment syndrome

CS can develop either due to extrinsic factors causing external compression or intrinsic factors causing internal expansion [8,21,22]. Extrinsic factors include casts, tight bandages, dressings, and burns. Intrinsic factors include inflammatory response, edema, hemorrhage, ischemia-reperfusion syndrome, and arterial pathology. Such factors are common in orthopedic surgery, patient positioning, and vascular trauma (Table 1).

**Table 1:** Perioperative factors responsible for developing compartment syndrome.

Event	Responsible factors
Following orthopedic fixation	Postoperative hematoma, muscle edema, or tight closure of the deep fascia
	It can be minimized by releasing the tourniquet before wound closure to ensure adequate hemostasis
Surgical positioning	Pressure-induced functional deficits due to decreased tissue perfusion rather than a direct mechanical effect
	The amount of pressure a limb can tolerate depends on limb elevation, blood pressure, hemorrhage, and arterial occlusion
	The mean time for the presentation is 15–24 hours or longer postoperatively
Vascular trauma	Reperfusion syndrome likely related to the ischemic depletion of high-energy phosphate forms and ischemic muscle injury

Risk factors

The development of CS depends on various risk factors, including patient type, nature of the injury, pharmacological and pathological factors, and surgical factors (Table 2). Age appears to be a strong predictor of developing ACS (p < 0.001), with the highest prevalence between 12-19 years and 20-29 years [23]. The reported incidence of ACS is 3.1 per 100,000 [24] and is ten times higher in men [25] younger than 35 [26]. Moreover, patients on anticoagulation medications are more prone to developing CS, especially in the thigh, even with minor trauma or surgical interventions. Development of ACS has been reported without any trauma in pediatric leukemia [3,13,14,16,27,28]. Such patients must be approached perioperatively with a high index of suspicion through regular clinical examinations (Table 3).

**Table 2:** Risk factors of compartment syndrome. CS: compartment syndrome

Categories	Risk factors
Patient category	Younger males (<35 years)
	Intense athletic activities (chronic CS)
	Patients with bleeding disorders
	Pediatric leukemia
Type of injuries	High-velocity injuries involving long-bone fractures
	Crush injuries
	Penetrating injuries (gunshot/stabbing)
	Vascular injuries (arteries/veins)
	Burns injuries
	Reperfusion injuries
Pharmacological factors	Drug overdoses
	Anticoagulation
Pathological factors	Thrombosis
	Infections
Surgical factors	Improperly placed casts or splints
	Tight circumferential bandages
	Poor positioning during surgery

**Table 3:** Clinical examination to look for signs of compartment syndrome.

	Clinical examinations
C:	Check for distal pulsations
O:	Observe for skin lesions, swelling, and color change
M:	Motor function evaluation
P:	Palpate affected limb for tightness and wood-like feeling
A:	Assess sensations
R:	Reassess for change in the pattern of sensation
T:	Test for temperature/tension/tenderness
M:	Measure compartment pressure
E:	Examine the unaffected limb and compare it with the affected limb
N:	Neurovascular examination
T:	Testing two-point discrimination and vibration sense

Symptoms

The chronology of symptoms can sometimes provide clues to the severity of CS, requiring a high level of suspicion during evaluations [29]. Any evidence of extensive trauma with or without gross deformity can alert the possibility of a developing CS. The timing of such symptoms also plays an important role in diagnosing CS severity. The occurrence of the symptoms depends on the rise in the compartment pressure and subsequent compression of the vasculature leading to ischemic changes in muscles, tissues, and nerves. ACS typically occurs within a few hours to 48 hours of inciting trauma. Classically, its presentation has been remembered by “The Five P’s”: pain, pulselessness, paresthesia, paralysis, and pallor [30,31]. Among these, paresthesia (tingling or burning sensations in the skin) may occur earlier than other symptoms [32] as the sensory nerves get affected before the motor nerves. The most consistent and reliable diagnostic finding or test is decreased two-point discrimination and sometimes diminished vibration sense (measured with 256 cycles/second tuning fork) [33]. The earliest objective/diagnostic physical finding of CS is the tense or “wood-like” feeling on deep palpation of the affected limb [34]. In comparison, objective evidence such as major sensorimotor deficit (numbness or paralysis) or loss of peripheral pulse suggests an advanced stage of CS due to compromised perfusion leading to permanent tissue injury.

The earliest clinical indicator of CS is typically severe pain disproportionate to injury or surgery, causing escalating analgesic demands. It occurs due to the release of inflammatory mediators of ischemic changes. The characteristics of pain in CS are burning, deep, and aching in nature, worsened by passive stretching of the involved muscles. However, pain may be absent in advanced ACS due to ischemic nerve damage.

Pathophysiology

**Figure 1:**
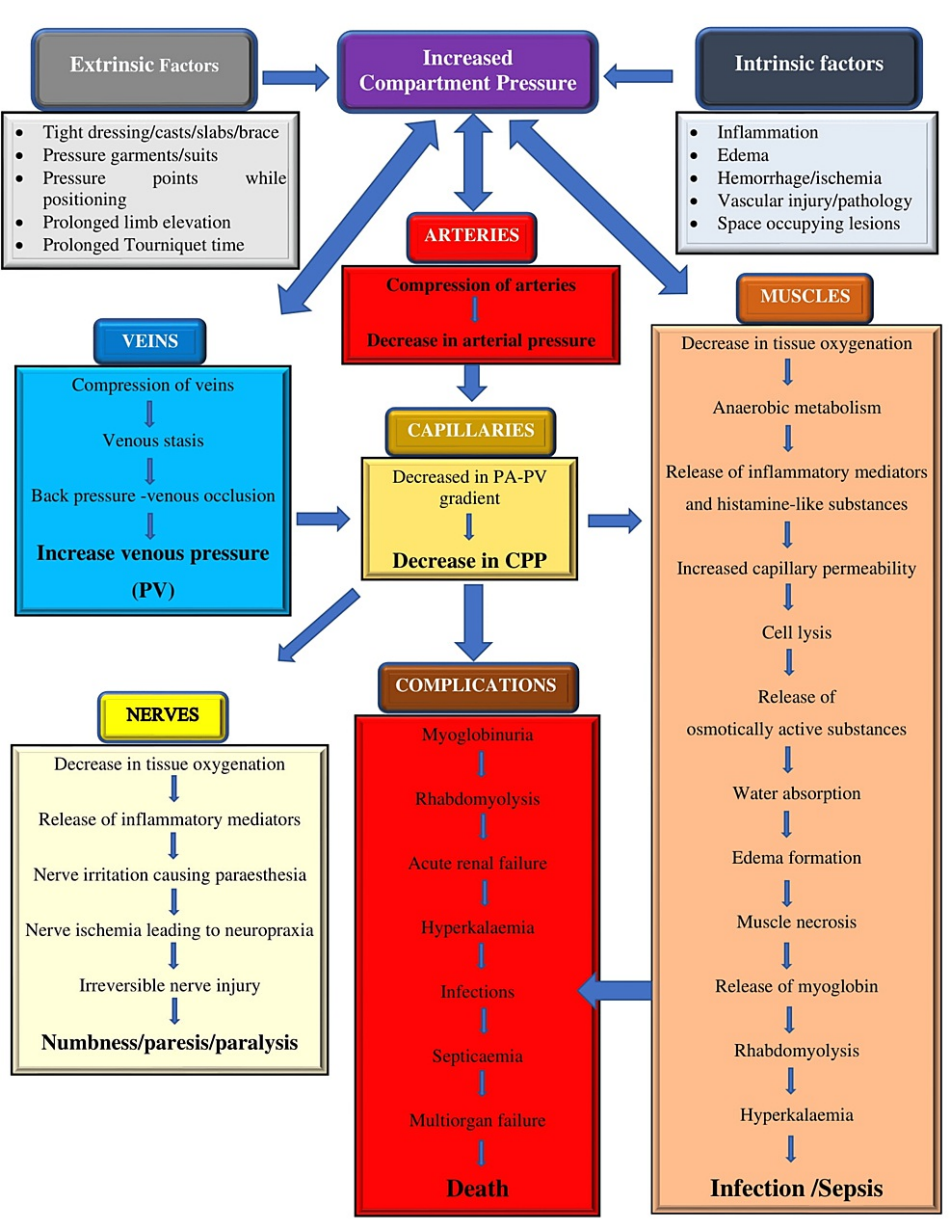
Pathophysiology of compartment syndrome. PV: venous pressure; CPP: capillary perfusion pressure Source: This figure was created by the first author (KS).

CS can develop predominantly from a significant rise in extraluminal or intraluminal pressure to the extent of causing impaired tissue perfusion, followed by hypoxia and ischemia [35]. Therefore, the first event leading to all subsequent events is the rise in compartment pressure due to external (extrinsic) or internal (intrinsic) factors, as described before. It is described either through the arteriovenous gradient theory or the ischemia-reperfusion syndrome [35]. The progressive increase in compartment pressure sequentially affects tissues, vessels, and nerves (Figure 1), leading to variations in the symptoms over time.

The rise in the compartment pressure first affects the venous outflow and later arterial inflow, disrupting the equilibrium between the two. Venous outflow reduction results in increased venous and capillary pressures, while arterial inflow reduction impairs oxygenation due to decreased tissue perfusion leading to irreversible necrosis. Tissue perfusion changes in the same direction as capillary perfusion pressure (CPP) and in the opposite direction as interstitial fluid pressure. The following formula explains it: LBF = (PA - PV)/R, where LBF is local blood flow, PA is local arterial pressure, PV is venous pressure, and R is local vascular resistance [36]. The metabolism of myocytes requires a CPP of 25 mmHg and interstitial fluid pressure of 4-6 mmHg [37]. Elevated tissue pressure reduces venous pressure narrowing arteriovenous (AV) perfusion gradient (PA-PV), resulting in capillary collapse and tissue/muscle ischemia.

Tissue/muscle ischemia causes the release of histamine-like substances that increase vascular permeability leading to plasma leakage and blood sludging in the small capillaries, aggravating ischemia [38]. Myocyte lysis due to ischemia leads to the degradation of the myofibrillar proteins into osmotically active particles. Such particles attract water from the arterial blood, further increasing intramuscular pressure [39]. Muscle ischemia and subsequent cellular edema worsen as tissue perfusion continues to decrease. This vicious cycle of deteriorating tissue perfusion continues.

Tissue tolerance to prolonged ischemia depends on the type of tissue affected: muscles have functional impairment initially after two to four hours, followed by irreversible functional loss after four to 12 hours [32], and nerves have an abnormal function after 30 minutes, followed by irreversible functional loss after 12 to 24 hours [40]. A significant change in the somatosensory potential can be seen as early as 45 minutes when the compartment pressure rises to 30 mmHg [40]. A sustained rise in the compartment pressure without treatment leads to ischemic necrosis of muscles and nerves with a limb contracture called Volkmann’s contracture. Severe cases may also lead to renal failure due to rhabdomyolysis and the release of myoglobin into the circulation after cell destruction and alterations in muscle cell membranes. Mortality can be associated with renal failure or sepsis due to difficult wound management [14,41-43].

Diagnosis

ACS is an emergency condition that requires prompt diagnosis as any delay in diagnosis and treatment can result in limb loss [44]. High-risk cases require regular inpatient monitoring for clinical features suggestive of CS (Table 3). Such cases include high-energy injuries, displaced fractures, or fibular fractures [45]. Despite limited sensitivity and specificity, the clinical features discussed above can help identify CS [34]. Measurement of intracompartmental pressure (ICP) has always been considered the gold standard for diagnosing ACS [46,47]. However, it is not always required but can aid in diagnosis when in doubt. It can be carried out with pressure-measuring devices such as manometers, tonometers, or transducers with slit catheters. Several handheld devices like the Stryker pressure tonometer are available [48-50]. With the slit catheter method, an arterial line transducer is connected to a catheter that can be inserted into the compartment to be measured. It is considered the most accurate method of measuring compartment pressures, allowing continuous monitoring. Serial and continuous ICP monitoring is recommended to avoid false-negative results associated with single normal ICP readings to confirm or exclude ACS [51-53]. Other less invasive techniques studied for measuring compartment blood flow include laser Doppler ultrasound, methoxy isobutyl isonitrile enhanced magnetic resonance imaging (MRI), phosphate-nuclear magnetic resonance (NMR) spectroscopy, thallous chloride-201 (201 Tl), and technetium-99 (99m Tc) sestamibi, and xenon (Xe) scanning [40].

As mentioned earlier, a single normal ICP measurement does not rule out ACS, which requires serial and continuous ICP monitoring. Measuring the perfusion pressure, also called the delta pressure (delta pressure = diastolic pressure - measured ICP) of the compartment, helps further evaluate and confirm the CS diagnosis [54]. The normal ICP is within 0-8 mmHg (<10-12 mmHg) [55-58]. Elevated ICP within 10-30 mmHg of the patient’s diastolic blood pressure indicates inadequate perfusion and relative ischemia of the affected limb. An ICP >30 mmHg or delta pressure <30 mmHg confirms the diagnosis of CS and indicates the need for fasciotomy [59]. Pain is common when the ICP reaches 20 mmHg, and an ICP >30 mmHg may be considered for emergency fasciotomy [25,60-63].

Laboratory workup (Table 4) can be needless for patients with classic presentation and physical findings of CS. Further, laboratory results do not help diagnose or rule out CS as they are often normal. Pulse oximetry helps detect limb hypoperfusion but is not sensitive enough to exclude CS. Near-infrared spectroscopy also aids in the non-invasive monitoring of oxygen saturation of hemoglobin and myoglobulin in tissue at risk [65]. Intramuscular pH monitoring has recently been introduced as an additional diagnostic tool to identify ACS accurately [66].

**Table 4:** Laboratory workups/imaging studies, and other investigations for compartment syndrome. CPK: creatine phosphokinase; CS: compartment syndrome; CT: computed tomography

Investigations	Significance
Complete blood count	Anemia can worsen oxygenation
Coagulation profile	To rule out disseminated intravascular coagulation, a rare but possible complication
CPK	The increased level may suggest muscle breakdown from ischemia, damage, or rhabdomyolysis
	CPK between 1,000 and 5,000 U/mL suggests CS
	Serial CPK measurements may show rising levels indicative of developing CS
Renal function and serum chemistry studies	Blood urea nitrogen and creatinine levels to assess hydration status
	Increased levels of K in rhabdomyolysis lead to fatal arrhythmias
Urinalysis	To rule out causes of acute renal failure
Urine myoglobin	The presence of urine myoglobin suggests CS
Radiographs/CT scanning	To determine the occurrence and the nature of fractures
Magnetic resonance imaging	To diagnose muscle tears as it may show increased signal intensity in an entire compartment on T2-weighted, spin-echo sequences [64]
Ultrasound with Doppler	To look for occlusion or thrombus
	To evaluate arterial flow and rule out deep venous thrombosis in lower extremities
	Loss of normal phasic patterns of tibial venous blood flow has been shown to accurately predict the need for surgical fasciotomy
Angiography	To determine vascular pathology or injuries
Muscle biopsies	To rule out primary muscle disorders
Histology	To rule out necrotizing fasciitis

Management of compartment syndrome

**Figure 2:**
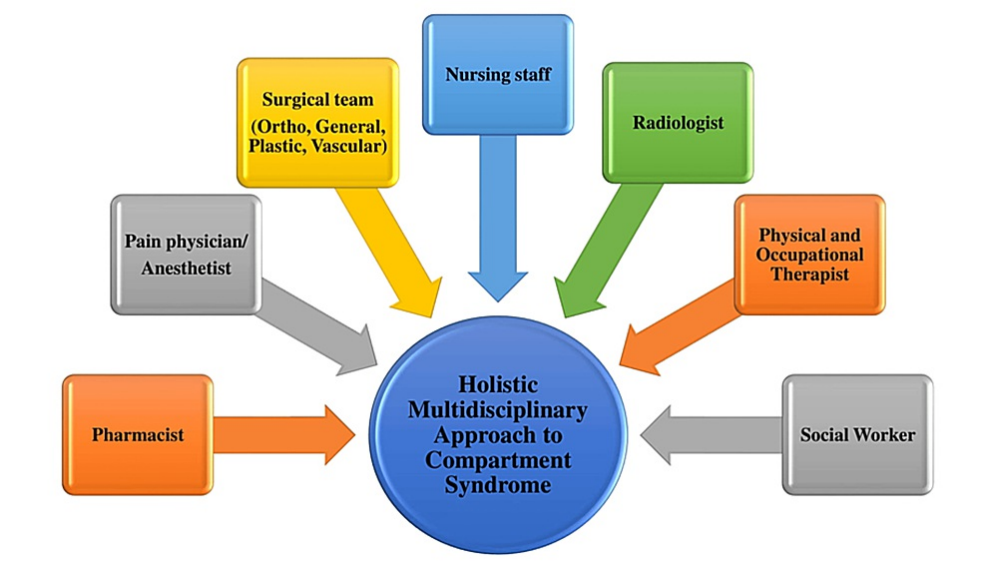
Multidisciplinary approach to compartment syndrome. Source: This figure was created by the first author (KS).

CS can lead to high morbidity and poor outcomes without proper management. The required complexity of the care can be provided through a holistic approach by a well-integrated interprofessional team of healthcare professionals (Figure 2), including surgical or orthopedic teams, nurses, physical therapists, occupational therapists, pharmacists, laboratory technologists, and social workers [67,68]. Nurses play a key role in the overall care of patients, beginning with the emergency room as the first medical professional to see the patient first through discharge. They can initiate care, quickly recognize signs and symptoms of CS, and involve the clinical team to evaluate it further. Along with other team members, they often play a role in coordinating care, performing procedures, and educating the patients/families about the nature of the injuries. Pharmacists can assist in medication reconciliation and pain management strategies. CS management includes prevention or arrest of the development of CS, treatment of developed ACS and its complications, and post-treatment care.

Prevention of compartment syndrome

Management strategies for prevention and arrest of the progress of CS can be decided by evaluating clinical symptoms and, if required, measuring ICP (Table 5).

**Table 5:** Management strategies to prevent or arrest the development of compartment syndrome. CS: compartment syndrome, ICP: intracompartmental pressure

ICP	Management strategies	
15–20 mmHg	Prevention of CS by doing uni-valving or bi-valving of the plaster cast to reduce the pressure by about 50%	
		20–30 mmHg	Immediate surgical consult	
Provide supplemental oxygen		
Remove any restrictive casts, dressings, or bandages to relieve pressure		
Keep the extremity at the level of the heart to prevent hypoperfusion. The elevation is contraindicated because it decreases arterial flow and narrows the arterial-venous pressure gradient [69,70]		
Prevent hypotension and correct hypoperfusion with the crystalloid solution and blood products		
ICP can be reduced by releasing one side of a plaster cast (30% reduction), bi-valving (additional 35% reduction), complete removal of the cast (another 15% reduction), and cutting undercast padding (10–30% reduction) [71-74]		
Relative hypertension and correction of acute anemia		
>30 mmHg	Immediate surgical fasciotomy	

Treatment of compartment syndrome

Fasciotomy

Early decompression of the compartment remains the treatment of choice for diagnosed CS. The emergent fasciotomy to restore muscle perfusion within six hours is the definitive surgical therapy that may follow fracture reduction or stabilization and vascular repair, if needed. A compartment pressure of 30 mmHg is considered the cut-off point for fasciotomy. However, multiple readings are often taken before making the final decision. Fasciotomy can be done within six hours of injury to avoid prolonged ischemic time leading to irreversible tissue damage. It is not recommended 36 hours after injury or is contraindicated after the third or fourth day following the onset of CS due to the lack of its benefit. However, if the duration of CS is unclear, the surgeon can decide on the decompression. A delayed fasciotomy can increase the risk of serious infection in the necrotic muscle. If left alone without opening the compartment, the necrotic muscle can heal with scar tissue, resulting in a more functional limb with fewer complications. Fasciotomy can lead to non-union and deep infections in approximately 20% of the patients [75]. Recommendations for performing fasciotomy [76] are included in Table 6.

**Table 6:** Recommendations for performing fasciotomy. ICP: intracompartmental pressure

ICP with duration	Patient profile
>20 mmHg	Hypotensive patient
>30 mmHg for unknown/>8 hour duration	Normotensive with positive clinical findings
>30 mmHg	Uncooperative and unconscious patient
30–40 mmHg for >4 hours	Normotensive with positive clinical findings
Or >40 mmHg	

In the context of a vascular injury, a fasciotomy should be performed in high-risk patients prior to arterial exploration. Such patients include those with prolonged ischemia time, significant preoperative hypotension, associated crush injury, combined arterial and venous injury, or the need for a major venous ligation in the popliteal or femoral area [40].

Post-fasciotomy

Ideally, wound closure after fasciotomy should be completed within one to five days or preceded by skin grafting for more than seven days [77]. After wound closure, additional surgical procedures such as tendon transfers and stabilization may be required. In addition to this, post-fasciotomy patients may require aggressive physical and occupational therapy to regain functions such as weight-bearing with assistive devices (e.g., crutches), followed by a rehabilitation program involving a range of motion (ROM), muscle flexibility, and adjacent joint exercises. In athletes, cross-training or sports-specific exercises/activities such as swimming, pedaling, water jogging, and running are initiated to return to a regular athletic schedule.

Medications

Pain management of patients with CS before and after fasciotomy is essential to improving the quality of care and ensuring patient comfort. Due to multifaceted pain-generating factors, the multimodal analgesic regimen is considered best in such conditions that include opioids and non-opioids (acetaminophen, non-steroidal anti-inflammatory drugs, and anticonvulsants like phenytoin, gabapentin, or carbamazepine). In addition to common pain relievers, newer remedies such as hyperbaric oxygen therapy (HBOT) or ultrafiltration have been considered. Prior treatment with HBO at the time of surgical debridement helps in delineating nonviable tissue due to its unique properties: it reduces swelling and edema by promoting hyperoxic vasoconstriction, it improves local blood flow and oxygenation, and it increases tissue oxygen tensions and improves the survival of marginally viable tissue [78,79]. The recommended dose of HBOT is 2.0 to 2.5-atmosphere absolute (ATA) over 90-120 minutes for five to seven days with frequent examinations of the affected area. On the other hand, ultrafiltration catheter insertion into the muscle compartment to monitor the biochemical environment within the tissue is being studied [80].

Treatment of the Complications

Patients with CS must be closely monitored for complications [10-11,81] such as chronic painful conditions, muscle contractures, rhabdomyolysis, nerve damage, infection, renal failure, and death. Muscle contractures occur due to muscle damage. Volkmann contracture can develop approximately in 1-10% of patients. It can occur over weeks to months following untreated ACS or ischemia from an uncorrected arterial injury [73]. Calcific myonecrosis is an uncommon late complication of post-traumatic CS. Recurrent CS reported in athletes is related to severe scarring and subsequent closing of the initial compartment release. Rhabdomyolysis, one of the life-threatening conditions, can be caused by muscle breakdown and death from overexertion, trauma, toxic substances, or disease. It involves the release of a muscle protein (myoglobin) into the circulation (myoglobinemia) and its excretion in the urine (myoglobinuria) through the kidneys. In large quantities, myoglobin can damage the kidneys. The combination of hypovolemia, acidemia, and myoglobinemia can lead to acute renal failure before or after surgery. Because hemoglobin and myoglobin are more soluble in an alkaline solution, alkalinization of the urine with bicarbonate and diuresis (to maintain urinary output at 1-2 mL/kg/hr) can be considered as a renal-protective treatment for rhabdomyolysis (Table 7) [40].

**Table 7:** Treatment for rhabdomyolysis. Source: This table was prepared by the first author (KS) from the information in reference number [40].

	Treatment
Aim	Alkalization of the urine
	Maintaining urine output at 1–2 mL/kg/hour
Correction of hypovolemia	500 mL/hour of crystalloid solution
Alkalization of the urine	22.4 mEq bicarbonate
Diuresis (if <300 mL/hour urine output)	Mannitol 1 g/kg
If blood pH >7.45	Acetazolamide 250 mg
Monitoring (hourly)	Vital signs, urine pH level, and volume
Assessments (six hourly)	Osmolarity, electrolytes, and arterial blood gas

Nerve damage is another bothersome complication that can result in numbness and/or weakness, hypesthesia, and painful dysesthesia that slowly resolves over time. Infection is a serious complication of CS that can become chronic following fasciotomy. It can lead to death if associated with metabolic complications of multiple traumatic injuries, multiorgan failure, and sepsis requiring prolonged intensive care [14,41].

Recommendations to avoid such complications include postoperative rehabilitation and physical therapy to regain function and strength and to prevent contractures and stiffness; wound care and monitoring for any ischemia, infection, or gangrene; antibiotics for possible infections and analgesics for pain; training to use an ambulatory device such as crutches until healing is complete; occupational therapy assisting the patient in performing activities of daily living [82-84].

Prognosis of compartment syndrome

The prognosis of the CS depends on its timely diagnosis and treatment. If diagnosed late, irreversible tissue ischemia can develop in the acute setting, leading to permanent muscle and nerve damage and chronic pain. If the fasciotomy is performed within six hours, limb function recovers almost 100% [85]. After six hours, the prognosis depends on the extent of damage caused by the ischemia affecting tissues and nerves [86]. Fasciotomy performed within 12 hours after the onset of ACS demonstrated normal limb function in only two-thirds (68%) of patients [87], and delayed fasciotomy >12 hours demonstrated normal limb function in only 8% of patients [40].

Long-term follow-ups after fasciotomies showed good results and a significant improvement in pain with a return to premorbid activity levels. However, some patients revealed residual pain, Volkmann’s contracture, mild neurological deficits, and significant cosmetic defects in the affected extremity. Very delayed cases may require limb amputation. There are chances of recurrent CS due to scarring, especially in athletes. The ease of decompression of the anterior compartment of the leg results in better outcomes than the posterior compartment. Death due to CS is often caused by infection, leading to sepsis and multiorgan failure [14,41-43]. Surviving patients almost always recovered from renal failures, including those on prolonged hemodialysis.

Regional anesthesia in compartment syndrome

In general, consideration of RA, especially in below-knee surgeries, is always a debatable factor due to the belief that it can mask clinical symptoms (mainly pain) that can delay the diagnosis and treatment of CS. For this reason, many centers prefer pain management without RA, mainly with systemic analgesics like opioids and non-opioids. However, how far can it be considered appropriate to rely completely on systemic analgesics to control postoperative pain and for that increasing dose and frequency of it? If pain is considered the sole diagnostic criterion, any effective analgesic protocol can lead to a delay in ACS diagnosis. Likewise, opioid-based pain management can also lead to a higher incidence of delay in diagnosis [88,89]. It is widely established that RA as an adjunct to multimodal analgesia has many benefits by reducing the frequency and dose of systemic analgesics and their subsequent side effects that can lead to delayed mobility and discharge. Apart from that, RA provides excellent postoperative analgesia and improves overall patient satisfaction. Most of the literature has focused primarily on pain as a cardinal symptom of ACS and held RA responsible for masking breakthrough pain and delaying ACS diagnosis. However, the analgesic method per se cannot be considered a reason for the delay in diagnosis [88,90] without focusing on the actual pathophysiology leading to pain generation following surgery, trauma, or subsequent ACS. The success of pain management lies in understanding pain pathology and differentiating expected postoperative pain from unexpectedly severe pain due to developing CS through vigilant periodic clinical assessments postoperatively. Moreover, depriving patients of their right to remain pain-free can be highly unethical.

Pain is a significant burden in the postoperative period that needs to be systematically addressed to avoid complications caused by the activation of cascades of pathways by undertreated or untreated pain. Postoperative pain mainly arises from inflammation limited to the dissected area. In contrast, the pain associated with CS involves both the ischemic and inflammatory pain pathways arising from dissected and non-dissected areas. However, both pathways involve tissue destruction leading to the release of numerous inflammatory mediators that cause continuous nociception in the affected area. Sustained production of these inflammatory mediators from the inflamed tissues stimulates free nerve endings, resulting in the generation of pain signals that are transmitted to the brain through spinal and supraspinal centers. The final pain perception at the central level depends on the trafficking of pain signals from the nociceptors. RA technique involves deposition of the LA around the nerves or plexuses that can block the pain conduction pathway by blocking the sodium channels. The interruption of the pain transmission to the brain affects the final central perception of the pain without affecting the ongoing inflammatory process at the periphery. The increased production of the inflammatory mediators due to ischemic tissue damage in ACS leads to hypersensitization of the nociceptors. The upregulation of the sodium channels (NaN/Nav1.9) [91,92] can lead to increased pain signal trafficking. The resultant hyperalgesia leads to increased analgesic demands even in the presence of dense RA block prior to the development of CS. Thus, ACS can be safely diagnosed [93] despite functional RA blockade. Therefore, given the risk of ACS, RA should not be contraindicated [94].

Some available literature blamed RA for the delayed ACS diagnosis [95-97]. In contrast, others favored RA by suggesting that it may not obscure a timely diagnosis [8,18,21,98-100] and may even facilitate early detection of ACS [101]. Interestingly, most of the available literature favoring or defending RA has been published in anesthesia journals and aversing the role of RA owing to delay in ACS diagnosis published in surgical/orthopedic journals [102]. The final decision on using RA remained ambiguous in an article in which the lead authors included surgical and anesthesiology faculties [103]. Many reports [21,99] highlighted unaffected ischaemic pain despite functional continuous regional blockade causing severe breakthrough pain. Almost all types of RA techniques have been reported to cause a delay in ACS diagnosis, including epidural analgesia [104], single-shot peripheral nerve blocks [10,105,106], and continuous PNB [21,99]. The most consistent feature in all these cases was the altered sensations in the affected limb [96], pain out of proportion in the presence of a functional nerve block, and escalating requirements of analgesics [99] that suggested underlying pathology rather than analgesic failure. The main symptom of the pain out of proportion to the surgery had been ignored in most of the case reports [98]. The escalating analgesic demands can significantly last for a longer duration (more than seven hours) in suspicious cases [107].

In most reported cases, the LA used was either bupivacaine or ropivacaine. The duration of the blockade of the pain pathway depends on the type, volume, and concentration of LA and the adjuvant used. Therefore, it should be seriously considered in high-risk patients as it directly affects the block quality [22]. Some literature recommends using diluted LA solution without any adjuvants in RA as part of MMA, particularly in patients with multiple comorbidities, to avoid not only CS but also high doses of opioids or systemic analgesics. Also, low concentrations of LA can facilitate the motor function of the limb and allow breakthrough pain detection [21,98,100,101]. LA, with its anti-inflammatory and anti-edema properties, may help prevent the development of CS [108,109]. All such scenarios prompt a need for a focused and regular clinical evaluation of the limb to look for warning signals suggestive of increased compartmental pressure [110].

## Conclusions

The diagnosis of CS is based solely on the clinical symptoms and sometimes the measurement of the ICP in suspected cases. Implementing RA should be encouraged as it can increase the blood flow through the sympathetic blockade without blocking the warning signs of ACS. Ultrasound guidance for RA and catheter insertion are strongly recommended to reduce LA dose and minimize the risk of delaying ACS diagnosis. More emphasis should be placed on developing a robust, valid, user-friendly, and reliable limb surveillance system for those at high risk of ACS rather than compromising with the analgesic method alone.
